# Transcriptomics reveals dynamic changes in the “gene profiles” of rat supraspinatus tendon at three different time points after diabetes induction

**DOI:** 10.1186/s12920-024-01899-3

**Published:** 2024-05-06

**Authors:** Kuishuai Xu, Liang Zhang, Tianrui Wang, Tengbo Yu, Xia Zhao, Yingze Zhang

**Affiliations:** 1 Department of Sports Medicine, The Affiliated Hospital of Qingdao University, Qingdao, 266000 Shandong China; 2 Department of Abdominal Ultrasound, Affiliated Hospital of Qingdao University, Qingdao, 266000 Shandong China; 3 Department of Traumatology, The Affiliated Hospital of Qingdao University, Qingdao, 266000 Shandong China; 4 Department of Orthopedic Surgery, Qingdao Municipal Hospital, Qingdao, 266000 Shandong China

**Keywords:** Transcriptomics, Type 2 diabetes, Tendinopathy, Signal path

## Abstract

**Objective:**

There is increasing evidence that type 2 diabetes mellitus (T2DM) is an independent risk factor for the occur of tendinopathy. Therefore, this study is the first to explore the dynamic changes of the “gene profile” of supraspinatus tendon in rats at different time points after T2DM induction through transcriptomics, providing potential molecular markers for exploring the pathogenesis of diabetic tendinopathy.

**Methods:**

A total of 40 Sprague-Dawley rats were randomly divided into normal (NG, *n* = 10) and T2DM groups (T2DM, *n* = 30) and subdivided into three groups according to the duration of diabetes: T2DM-4w, T2DM-8w, and T2DM-12w groups; the duration was calculated from the time point of T2DM rat model establishment. The three comparison groups were set up in this study, T2DM-4w group vs. NG, T2DM-8w group vs. NG, and T2DM-12w group vs. NG. Differentially expressed genes (DEGs) in 3 comparison groups were screened. The intersection of the three comparison groups’ DEGs was defined as key genes that changed consistently in the supraspinatus tendon after diabetes induction. Cluster analysis, gene ontology (GO) functional annotation analysis and Kyoto encyclopedia of genes and genomes (KEGG) functional annotation and enrichment analysis were performed for DEGs.

**Results:**

T2DM-4w group vs. NG, T2DM-8w group vs. NG, and T2DM-12w group vs. NG detected 519 (251 up-regulated and 268 down-regulated), 459 (342 up-regulated and 117 down-regulated) and 328 (255 up-regulated and 73 down-regulated) DEGs, respectively. 103 key genes of sustained changes in the supraspinatus tendon following induction of diabetes, which are the first identified biomarkers of the supraspinatus tendon as it progresses through the course of diabetes.The GO analysis results showed that the most significant enrichment in biological processes was calcium ion transmembrane import into cytosol (3 DEGs). The most significant enrichment in cellular component was extracellular matrix (9 DEGs). The most significant enrichment in molecular function was glutamate-gated calcium ion channel activity (3 DEGs). The results of KEGG pathway enrichment analysis showed that there were 17 major pathways (*p* < 0.05) that diabetes affected supratinusculus tendinopathy, including cAMP signaling pathway and Calcium signaling pathway.

**Conclusions:**

Transcriptomics reveals dynamic changes in the“gene profiles”of rat supraspinatus tendon at three different time points after diabetes induction. The 103 DEGs identified in this study may provide potential molecular markers for exploring the pathogenesis of diabetic tendinopathy, and the 17 major pathways enriched in KEGG may provide new ideas for exploring the pathogenesis of diabetic tendinopathy.

## Introduction

Tendinopathy is commonly seen after degenerative changes in the tendons, often characterized by pain and limited movement, and its pathogenesis is still unclear [1]. In recent years, relevant studies have shown that diabetes is closely related to tendon injury, and diabetes increases the risk of tendon injury. Longo et al. [2] retrospectively analyzed 194 patients who underwent arthroscopic surgery from 2007 to 2008, and found that patients with diabetes were more likely to have rotator cuff injury, and hyperglycemia was a risk factor for rotator cuff injury (*P* = 0.007). Spoendlin et al. [3] divided 7,895 patients with achilles tendon injury or biceps tendon injury from 1995 to 2013 into diabetic group and non-diabetic group, and found through comparative analysis that the degree of control of blood sugar level in female patients was closely related to tendon injury. Park et al. [4] selected 634 subjects diagnosed with rotator cuff tears and found that diabetes was one of the risk factors for rotator cuff tears. Other studies have found that the likelihood of tendinopathy in patients with diabetes is 4 times that of nondiabetic, and the likelihood of tendon tear or rupture is 5 times that of nondiabetic [5].

Previous studies have confirmed that diabetes promotes tendon degeneration and promotes the occurrence of tendinopathy. Batista et al. [6] found that 62 of the 70 patient with diabetes had tendon fiber disorder and 17 had tendon calcification through color ultrasound. Another study [7] conducted shoulder joint ultrasound examination on 48 elderly patient with diabetes without shoulder pain or dysfunction and 32 control patients. The results showed that the mean thickness of supraspinatus muscle tendon in patient with diabetes was 6.2 mm, which was higher than 5.2 mm in control group (*P* < 0.05). At the same time, the results of ultrasound examination showed that 42.7% of patient with diabetes with supraspinatus tendon degenerative changes, much higher than the control group. All the above experiments showed that diabetes was closely related to the occurrence of tendinopathy. Diabetes damages the normal structure of tendons from many aspects such as tendon collagen, blood vessels and nerves, and then changes the biomechanical properties of tendons. However, many of the specific influencing mechanisms are still unclear and need more in-depth research.

A comprehensive understanding of the variations occurring in diseased tendons is essential to prevent the onset and progression of tendinopathy and to relieve symptoms. Due to continuous technological innovation and continuous cost reduction, transcriptomic sequencing has been increasingly used as a powerful alternative to bulk RNA sequencing over the past few years. Transcriptomics is based on Illumina sequencing platform to study all mRNA transcribed by a specific tissue or cell ina certain period, which is the basis of gene function and structure research, and plays an important role in understanding the development of organisms and the occurrence of diseases. To date, transcriptome changes in injured tendons have been reported in several studies using bulk RNA sequencing [8–10]. In addition, single-cell and spatial RNA sequencing methods were used to further understand the cellular heterogeneity and molecular mechanism of tendinopathy progression, and the study results provided new insights into the control of tendinopathy [11]. Single-cell and spatial transcriptomics have shown that the pathogenesis of human tendinopathy is closely related to dysregulation of immune homeostasis [12]. Multi-omics techniques have been widely used to explore the pathogenesis of tendinopathy, but until now, transcriptomic techniques have not been applied to the study of diabetic tendinopathy.

In previous studies, we successfully established an animal model of rotator cuff tendinopathy in diabetic rats by comparing the histological and biomechanical changes of supraspinatus tendon at 2,4,8 and 12 weeks after diabetes induction [13]. More and more studies have explored the changes of gene transcription in diabetes-related complications through transcriptomics technology, and most of them have used rat models as experimental objects [14–17], so the rat model can be used as experimental objects close to the human model. In this study, we collected tendons of the supraspinatus tendon from T2DM and normal rats and then performed transcriptome sequencing. By constructing the of the supraspinatus muscle tendon of diabetic rats, we found the dynamic change of the “gene profile” in the occur of rotator cuff tendinopathy with the progression of diabetes, and found the key genes and potential pathogenic pathways that may be closely related to the pathogenic mechanism. These findings will help us to more thoroughly understand the pathogenesis of diabetes leading to supraspinatus tendinopathy and provide potential targets for the treatment of diabetic tendinopathy.

## Methods

### Experimental animals

SPF-grade healthy male Sprague-Dawley rat, aged 8 weeks and weighing 220 ∼ 250 g, was provided by Animal Research Center of Qingdao University. Animal License Number: SCXK (Zhejiang) 2019-0001. This experiment was approved by the Animal Ethics Committee of Qingdao University (Approval No. 20220505SD8020221210126). The study is reported in accordance with ARRIVE guidelines.

### Animal grouping

A total of 40 Sprague-Dawley rats were randomly divided into normal (NG, *n* = 10) and type 2 diabetes mellitus groups (T2DM, *n* = 30) and subdivided into three groups according to the duration of diabetes: T2DM-4w, T2DM-8w, and T2DM-12w groups; the duration was calculated from the time point of T2DM rat model establishment. High-fat diet(#MD12033; MediScience Diets Co., Ltd., Yangzhou, China)was fed for 2 weeks. The rats were housed in clean conditions with temperatures maintained at 20–24 °C. All rats ate 3 regular meals with 12 h of light per day, and turned off the light and stopped providing food at night. After 2 weeks, rats were deprived of water for 12 h and intraperitoneally injected with Streptozotocinn (STZ; Aladdin Co., Ltd., Beijing, China). After 72 h, tail venous blood was taken to detect fasting blood glucose of rats [18]. If the blood glucose level was ≥ 16.7 mmol/l for 3 consecutive days, the diabetic rat model was considered to be successfully established [19]. The normal group was fed with ordinary diet for 2 weeks, and after 2 weeks, the same amount of sodium citrate buffer ( BIOISCO Co., Ltd., Nanjing, China).

### Obtain sample

At the 4th, 8th and 12th week after modeling, the bilateral supraspinatus muscle of diabetic rats was completely dissected, the excess muscle tissue around the tendon was separated, and the complete supraspinatus tendon was obtained, rinsed with phosphate-buffered saline (PBS), and the tissue samples were stored in a -80°refrigerator after drying. The tendons of supraspinatus muscle of normal rats were taken by the same method. In order to meet the requirements of transcriptomics for sample quality, supraspinatus tendon tissues of two animals (four shoulders) were combined to construct a transcriptomic sample.

### RNA extraction and bioinformatics analysis of RNA-seq

Total RNA was isolated and purified using Trizol reagent (Invitrogen, Carlsbad, CA, USA) following the manufacturer’s procedure. The RNA amount and purity of each sample was quantified using NanoDrop ND-1000 (NanoDrop, Wilmington, DE, USA). After quality control, libraries were constructed as previously described [20]. mRNA-seq were performed on the Hiseq4000 platform (Illumina, CA, USA) with 150 strategy. The method of RNA-seq bioinformatics analysis was consistent with previous studies [21]. The differentially expressed mRNAs were selected with fold change>2 or fold change<0.5 and with parametric F-test comparing nested linear models (adjusted p-value<0.05) by R package edgeR (https://bioconductor.org/packages/release/bioc/html/edgeR.html). Sample processing, computer sequencing and data visualization analysis were all assisted by Lc-Bio Technologies (Hangzhou, China).

## Results

### Three comparison groups of DEGs

**Fig. 1:**
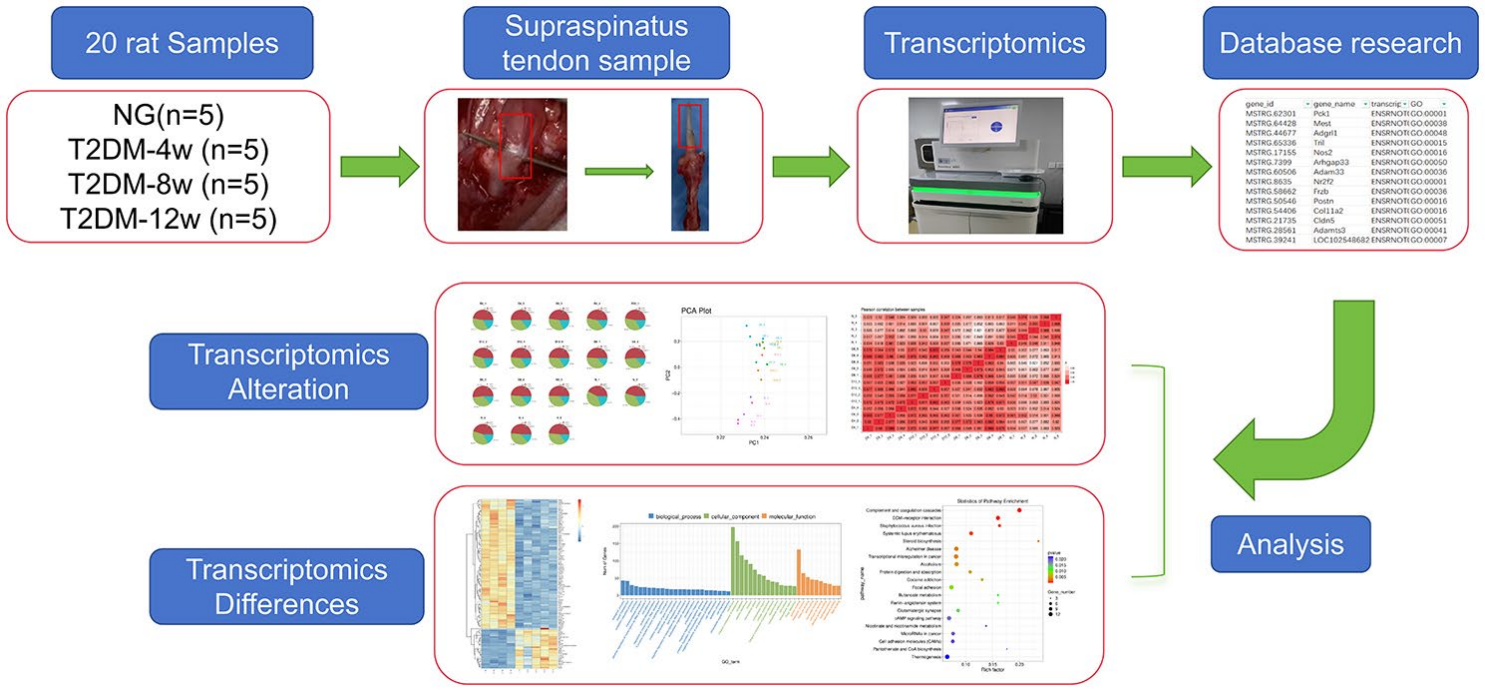
Study design and transcriptomics analysis of the supraspinatus tendon in diabetic rats. Overview of the cohort (including 5 normal samples, 5 type 2 diabetes 4-week samples, 5 type 2 diabetes 8-week samples, and 5 type 2 diabetes 12-week samples) and study design (including RNA extraction, library construction, bioinformatics analysis of RNA-seq, and screening of DEGs)

**Fig. 2:**
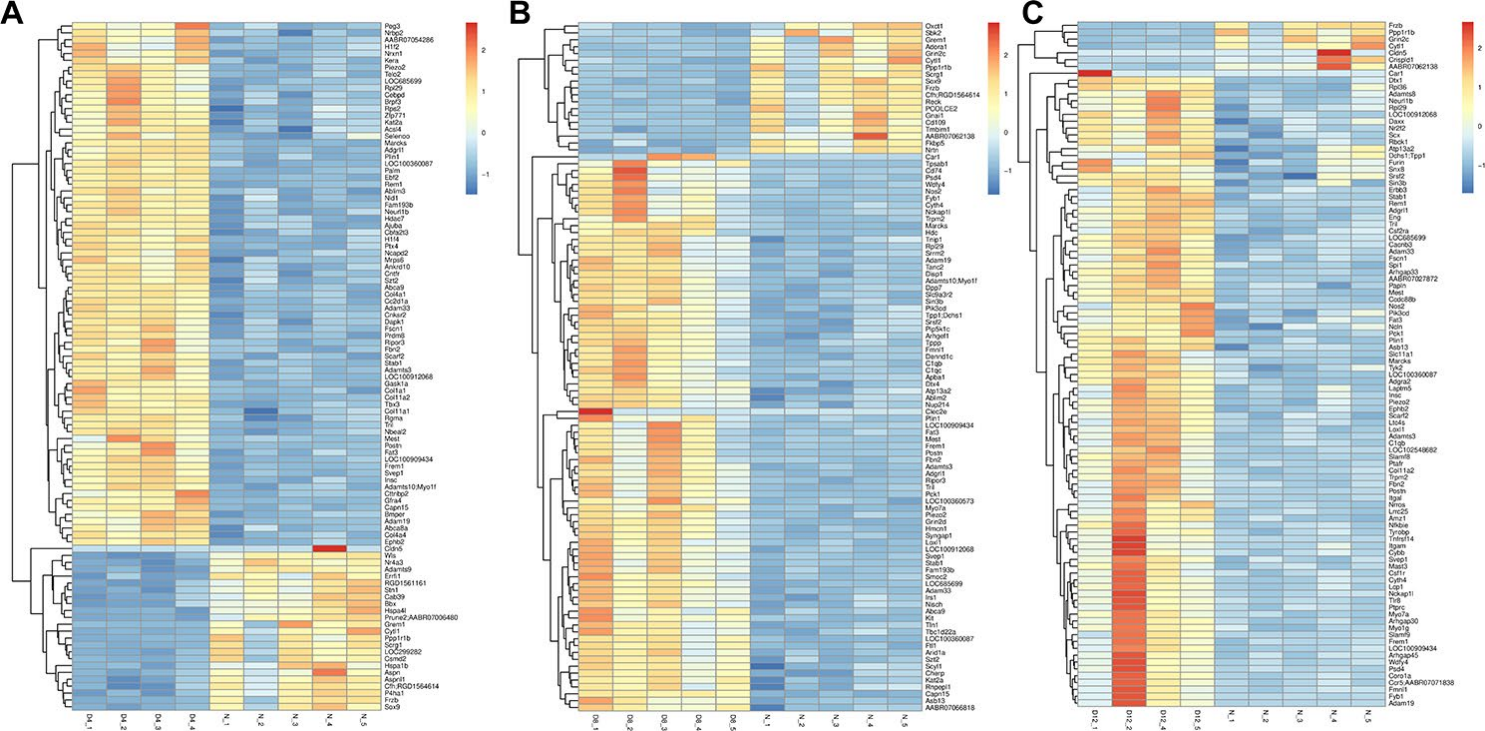
Heatmap of annotated genes with increasing and decreasing trend, including T2DM-4w group vs. NG (**A**), T2DM-8w group vs. NG (**B**), and T2DM-12w group vs. NG (**C**). Each column represents a sample, and each gene is visualized in a row red indicates a high abundance, and blue indicates a relatively low abundance of genes

**Fig. 3:**
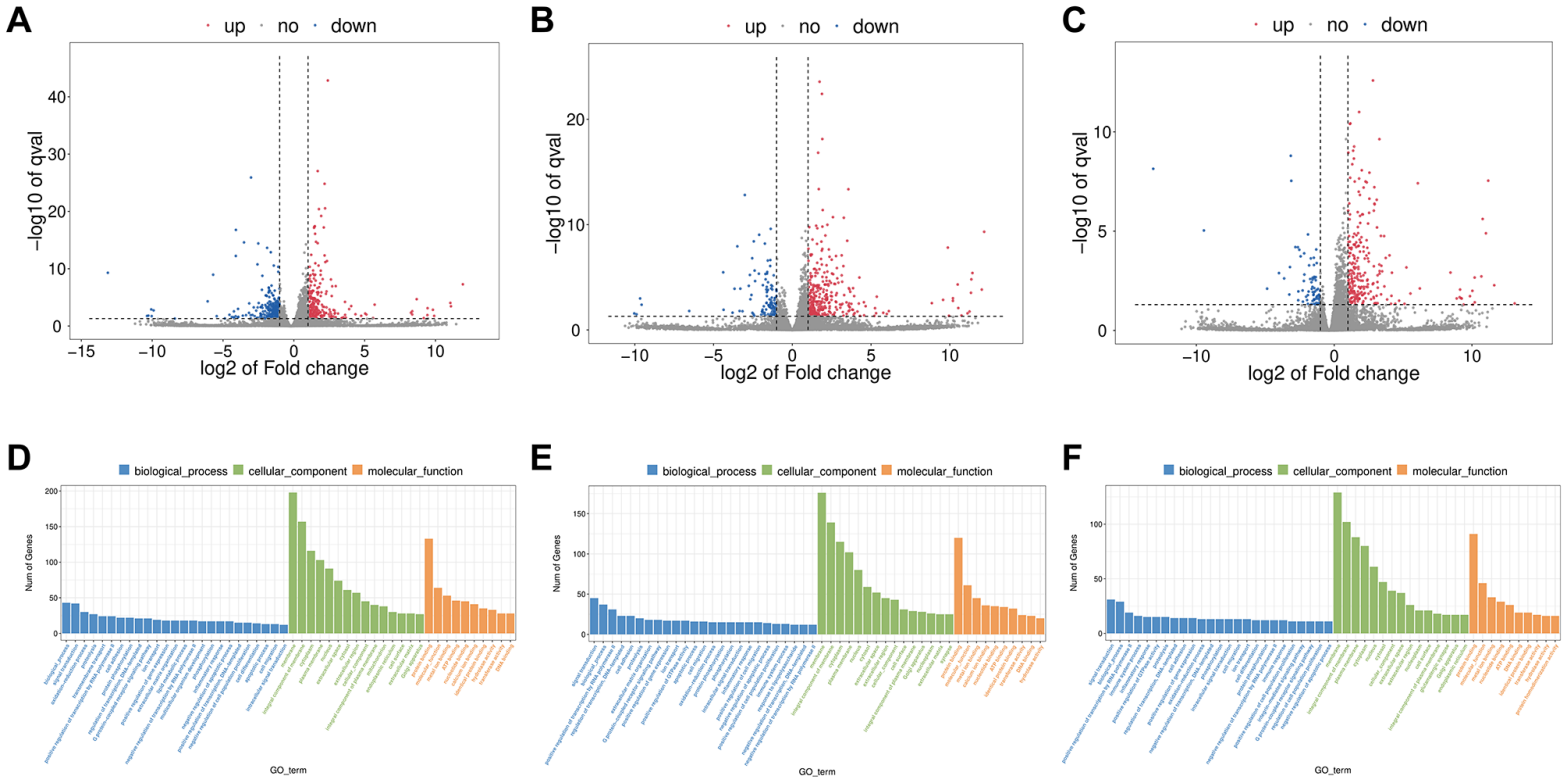
Volcano plot of DEGs between three comparison groups. Each dot represents one gene red dots represent the significantly upregulated genes, and blue dots represent the significantly downregulated genes gray dots represent no significant DEGs. (**A**) T2DM-4w group vs. NG: (**B**) T2DM-8w group vs. NG: (**C**) T2DM-12w group vs. NG. According to the principle of Gene ontology (GO) analysis, the biological function of genes is divided into three main parts, namely biological process (BP), cellular component (CC) and molecular function (MF). (**D**) T2DM-4w group vs. NG: (**E**) T2DM-8w group vs. NG: (**F**) T2DM-12w group vs. NG

20 supraspinatus tendon samples were extracted from normal and diabetic rats in this study to reveal a list of genes associated with the occur of diabetic tendinopathy (Fig. 1). In the process of data analysis, we found that two samples had little correlation with other samples in the group, and we eliminated these two samples. Therefore, the T2DM-4w and T2DM-12w groups are 4 samples. In order to determine the effect of T2DM on the transcriptome of supraspinatus tendon in rats and its dynamic change process, the “gene profile” of supraspinatus tendon was compared 4, 8 and 12w after T2DM induction and in the control group. T2DM-4w group vs. NG, T2DM-8w group vs. NG, and T2DM-12w group vs. NG detected 519 (251 up-regulated and 268 down-regulated), 459 (342 up-regulated and 117 down-regulated) and 328 (255 up-regulated and 73 down-regulated) DEGs, respectively. The upregulated and downregulated DEGs identified in the 3 comparison groups were ranked in order of change multiples from largest to smallest, and the top 10 DEGs induced by T2DM were listed (Table 1). Cluster analysis was performed on DEGs of the three comparison groups. The cluster heat map (Fig. 2) and volcano map (Fig. 3A-C) clearly showed the distribution of DEGs at different time points after T2DM induction.

**Table 1:** Three comparison groups up-regulated and down-regulated DEGs (Top10)

Grouping situation	Up-regulated (Top10)		Down-regulated (Top10)	
	DEGs	FC	DEGs	FC
T2DM-4w vs. NG	Gfra4	3897.56	Cldn5	0.00
	Ccl24	2176.51	Olr1158	0.00
	Tpsab1	2116.26	Mmp10	0.00
	Serpina12	959.17	Btnl8	0.00
	Prr35	900.64	Ibsp	0.00
	Capn13	693.56	Clec2dl1	0.00
	Tmem156	687.61	Glrb	0.00
	Hoxb5	576.23	Pcsk2	0.01
	Asxl3	407.09	Grem1	0.02
	Mrgprx2	327.92	Crtac1	0.02
T2DM-8w vs. NG	Car1	4638.35	Ibsp	0.00
	Fcgr3a	4173.02	Nxph4	0.00
	Tpsab1	2769.46	Crtac1	0.00
	Gfra4	2636.00	Pkd2l1	0.00
	Tnfrsf14	2593.24	AABR07026004	0.01
	AABR07027872	2434.26	Grem1	0.05
	RGD1560958	2228.16	Krt15	0.05
	RGD1565785	1707.26	Gad2	0.07
	Trem3	1484.39	Vat1l	0.08
	LOC689757	1445.51	Ppp1r1b	0.09
T2DM-12w vs. NG	RT1-Ba	8748.25558	Cldn5	0.000111689
	AC128059	3132.631182	Crispld1	0.001428294
	Car1	2321.5075	Col2a1	0.034305661
	AABR07027872	2063.91	Grem1	0.062328619
	Tnfrsf14	1749.1375	Ucma	0.080858304
	Gfra4	1643.9375	Pcsk2	0.10500524
	Tpsab1	1175.7325	Vat1l	0.108438803
	RGD1565785	1169.89	Frzb	0.112909693
	Clec4a2	1066.46	Grin2c	0.115684057
	Pdcd1	1043.0575	Ppp1r1b	0.142230161

### GO enrichment analysis

In order to study the potential biological functions and pathways of tendinopathy caused by diabetes, GO enrichment analysis of these DEGs was further conducted (Fig. 3D-F). According to the principle of GO analysis, the biological function of genes is divided into three main parts, namely biological process, cellular component and molecular function. In the comparison of T2DM-4w and NG, and the biological processes, cellular component and molecular function enrichment of DEGs are biological process, membrane and protein binding, there are 78, 198, 133 DEGs, respectively. In comparison of T2DM-8w and NG, and the biological processes, cellular component and molecular function enriched DEGs were the signal transduction, membrane and protein binding, there are 45, 176 and 120 DEGs, respectively. In the comparison of T2DM-12w and NG, and the biological processes, cellular component and molecular function enriched DEGs were the signal transduction, membrane and protein binding, there were 31, 129 and 91 DEGs, respectively. In summary, compared with NG, with the progression of diabetes, DEGs in T2DM group was mainly enriched in signal transduction, membrane and protein binding.

### KEGG annotation classification and pathway enrichment

**Fig. 4:**
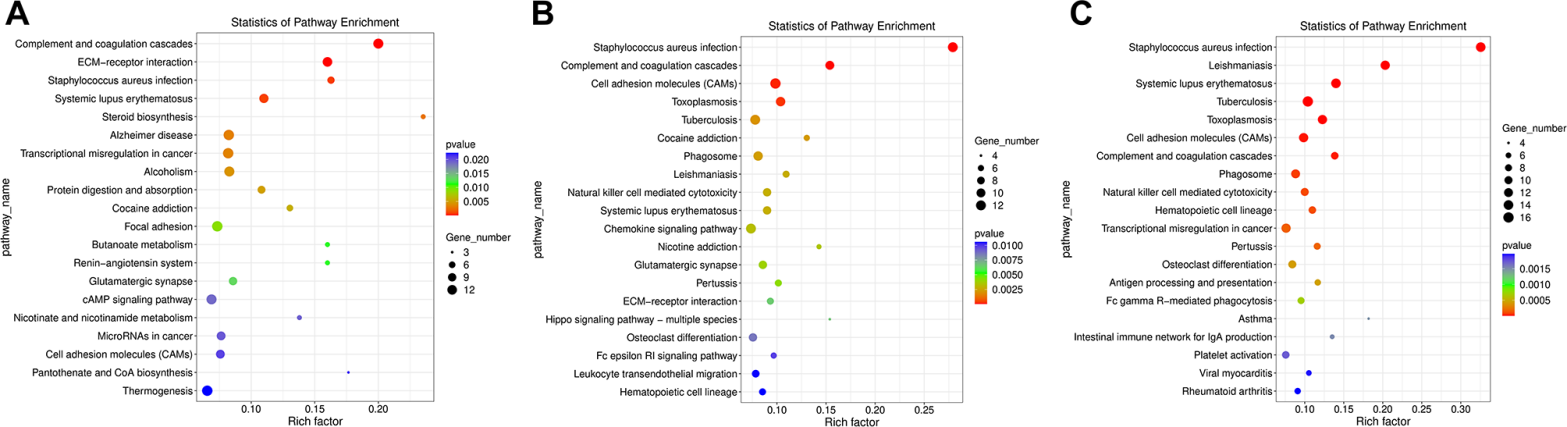
The KEGG functional enrichment analysis in three comparison groups. (**A**) T2DM-4w group vs. NG: (**B**) T2DM-8w group vs. NG: (**C**) T2DM-12w group vs. NG

KEGG database was used for functional annotation classification and pathway enrichment of DEGs (Fig. 4). In the comparison of T2DM-4w and NG, KEGG pathway enrichment analysis results showed that the first three affected pathways included ECM-receptor interaction, cAMP signaling pathway, and PI3K-Akt signaling pathway showed higher enrichment and number of differential genes. In the comparison of T2DM-8w and NG, KEGG pathway enrichment analysis results showed that the first three affected pathways included ECM-receptor interaction, Fc epsilon RI signaling pathway, and Rap1 signaling pathway had higher enrichment degree and number of differential genes. In the comparison of T2DM-12w and NG, KEGG pathway enrichment analysis results showed that the first three affected pathways included B cell receptor signaling pathway, cAMP signaling pathway, and Chemokine signaling pathway had higher enrichment degree and number of differential genes.

### Transcriptomics alterations associated with the occur of diabetic tendinopathy

**Fig. 5:**
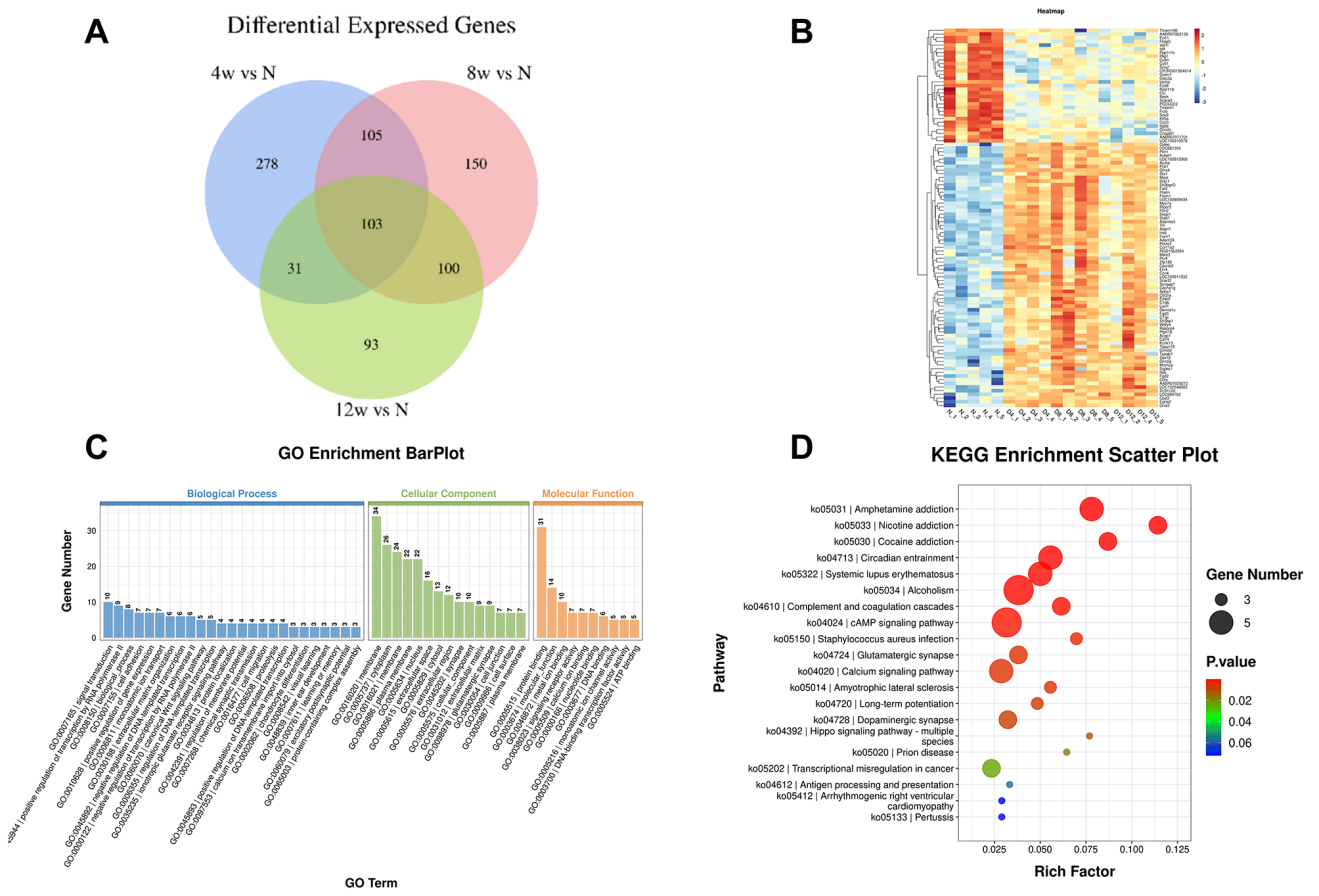
(**A**) The Venn diagram compares the number of different genes among all three comparison groups, including the paired comparison groups of T2DM-4w group vs. NG, T2DM-8w group vs. NG, and T2DM-12w group vs. NG. (**B**) Clustering heatmap of 103 DEGs. (**C**) Gene ontology (GO) enrichment analysis. (**D**) KEGG pathway analysis

The result of the Venn diagram shows 103 key genes of sustained changes in the supraspinatus tendon following induction of diabetes, which are the first identified biomarkers of the supraspinatus tendinopathy as it occurs through the course of diabetes (Fig. 5A). The cluster heat map (Fig. 5B) clearly showed the distribution of DEGs at different time points after T2DM induction. The GO analysis results (Fig. 5C) showed that the most significant enrichment in biological processes was calcium ion transmembrane import into cytosol (3 DEGs). The most significant enrichment in cellular component was extracellular matrix (9 DEGs). The most significant enrichment in molecular function was glutamate-gated calcium ion channel activity (3 DEGs). The biological processes, cellular component and molecular function enriched DEGs were the signal transduction, membrane and protein binding, there were 10, 34 and 31 DEGs, respectively. The results of KEGG pathway enrichment analysis showed that there were 17 major pathways (*p* < 0.05) that diabetes affected supratinusculus tendinopathy, including cAMP signaling pathway and Calcium signaling pathway Fig. 5D; Table 2.

**Table 2:** Pathways of major enrichment of 103 critical genes (*p* < 0.05)

Pathway ID	Pathway description	DEGs	*p*-value
ko05031	Amphetamine addiction	Grin2a, Ppp1r1b, Grin2c, Gria3, Grin2d	4.93988E-05
ko05033	Nicotine addiction	Grin2a, Grin2c, Gria3, Grin2d	6.67289E-05
ko05030	Cocaine addiction	Grin2a, Ppp1r1b, Grin2c, Grin2d	0.000197593
ko04713	Circadian entrainment	Grin2a, Cacna1g, Grin2c, Gria3, Grin2d	0.00025181
ko05322	Systemic lupus erythematosus	Grin2a, LOC102548682, LOC684762, C1qb, C1qc	0.000411452
ko05034	Alcoholism	Grin2a, Ppp1r1b, Grin2c, LOC102548682, LOC684762, Grin2d	0.000447412
ko04610	Complement and coagulation cascades	Cfh; RGD1564614, Clu, C1qb, C1qc	0.000750526
ko04024	cAMP signaling pathway	Grin2a, Ppp1r1b, Sox9, Grin2c, Gria3, Grin2d	0.001188997
ko05150	Staphylococcus aureus infection	Cfh; RGD1564614, C1qb, C1qc	0.002545926
ko04724	Glutamatergic synapse	Grin2a, Grin2c, Gria3, Grin2d	0.004402465
ko04020	Calcium signaling pathway	Grin2a, Cacna1g, Grin2c, Grin2d, Erbb3	0.004833714
ko05014	Amyotrophic lateral sclerosis	Grin2a, Grin2c, Grin2d	0.004872645
ko04720	Long-term potentiation	Grin2a, Grin2c, Grin2d	0.007170311
ko04728	Dopaminergic synapse	Grin2a, Ppp1r1b, Gria3.Kif5a	0.007913662
ko04392	Hippo signaling pathway - multiple species	Ajuba, LOC100909434	0.01187967
ko05020	Prion disease	C1qb, C1qc	0.016656696
ko05202	Transcriptional misregulation in cancer	Etv4, Gria3, LOC102548682, LOC684762	0.023364893

## Discussion

To date, transcriptome changes in injured tendons have been reported in several studies using bulk RNA sequencing [8–10]. In addition, single-cell and spatial RNA sequencing methods were used to further understand the cellular heterogeneity and molecular mechanism of tendinopathy progression, and the study results provided new insights into the control of tendinopathy [11]. Single-cell and spatial transcriptomics have shown that the pathogenesis of human tendinopathy is closely related to dysregulation of immune homeostasis [12]. At present, more and more studies are trying to discover the potential pathogenic small molecules of diabetes mellitus and its related complications through transcriptomics. Transcriptomics has a wide range of applications, including diabetic nephropathy, atherosclerosis, and retinopathy [22–24], which has resulted in the accumulation of large amount of biological data. However, the alterations in supraspinatus muscle tendon genes and the signaling pathways involved in their role in diabetic rats are still unclear.

This is the first study to establish a list of biomolecules that may be involved in the dynamic changes of the supraspinatus tendon of the rotator cuff with the progression of diabetes using transcriptomic techniques. In addition, we identified 103 key genes that may be closely related to the development of rotator cuff tendinopathy, and KEGG enrichment to 17 pathways, including the cAMP signaling pathway and Calcium signaling pathway. These results may provide a new target and theoretical basis for exploring the pathogenesis of diabetic tendinopathy.

The study contains a wealth of biological information that other researchers can use to explore the molecular changes associated with diabetes and tendinopathy. At the same time, KEGG analysis identified novel pathways that are differentially regulated in diabetic tendinopathy, including the cAMP signaling pathway and Calcium signaling pathway. Previous studies have shown that Cyclic adenosine monophosphate (cAMP) is an important intracellular second messenger, which plays various physiological roles through the activation of protein kinase A(PKA), and is a key regulatory factor in regulating metabolism and maintaining glucose homeostasis [25]. cAMP is known to play an important regulatory role in a variety of important physiological and pathological processes, including endothelial angiogenesis [26], osteoclast function [27], and reduction of oxidative stress [28]. cAMP signaling pathway also plays an important role in various complications caused by diabetes. cAMP signaling pathway is one of the important pathways in anti-diabetic nephropathy with renal fibrosis. Deb et al. [29] confirmed that activation of cAMP signaling pathway is an important mechanism to induce the transformation of renal tubular epithelial cells into stromal cells, thus promoting the progression of renal fibrosis. The activation of cAMP signaling pathway is closely related to increased glucose uptake in skeletal muscle. This physiological process is independent of insulin regulation, and the regulation of cAMP signaling pathway in skeletal muscle can be used as an alternative pathway for glucose processing and blood sugar control [30].

Of course, cAMP has a stimulating effect on insulin secretion and plays an active coordinating role in glucose-stimulated insulin secretion [31]. Hellman and Grill found that increased cAMP concentration plays an important role in glucose-stimulated insulin secretion in beta cells [32, 33]. Intracellular Ca^2+^ elevation is considered to be the ultimate activation mechanism of insulin secretion by beta cells. Later studies confirmed that glucose stimulation increases the level of cAMP, which is related to Ca^2+^ concentration [34, 35]. Our study also found that calcium signaling pathway may be associated with the development of diabetic tendinopathy, but whether there is a correlation between the two pathways requires further investigation of the mechanism. In addition, protecting islets by targeting cAMP signaling pathway is also an important strategy to promote glucose homeostasis. Because of the key role of cAMP signaling in regulating physiological and pathological processes, many drugs targeting cAMP are used in various diseases [36–39]. However, the correlation between cAMP signaling pathway and tendinopathy has not been reported. From the perspective of interfering with cAMP signaling pathway, it may be a new idea to actively develop prevention and treatment drugs to inhibit diabetic tendinopathy.

In addition, we found that the mechanism of tendinopathy induced by T2DM may be related to the PI3K-Akt signaling pathway and AMPK signaling pathway. Previous literature has also confirmed that the PI3K-Akt signaling pathway is closely related to impaired wound healing related to diabetes, and the up-regulation of the PI3K/Akt signaling pathway may be a promising target for accelerating healing [40]. AMPK and PI3K/Akt signaling pathways may be involved in diabetes-induced cognitive decline, and DL-3-n-butylphthalide ameliorates diabetes-associated cognitive decline by enhancing PI3K/Akt signaling [41].AMPK may be a fundamental cellular pathway for metabolic disorders and diabetes [42]. AMPK-mediated inflammation and oxidative stress are associated with decreased angiogenesis of endothelial progenitor cells associated with diabetes [43]. Meanwhile, AMPK and PI3K/Akt signaling pathways are closely related to mediating autophagy [44]. Diabetes is considered to be a mitochondrial disease at least to a certain extent, and because mitochondria play an important role in maintaining environmental homeostasis within rotator cuff tendons, mitochondrial damage may be associated with the occurrence of diabetic tendinopathy. However, whether autophagy mediated by AMPK and PI3K/Akt signaling pathways is involved in diabetes-induced rotator cuff tendinopathy is unclear. In the future, more work is needed to validate the role of AMPK and PI3K/Akt signaling pathways in diabetic tendinopathy.

In conclusion, the “gene profile” of supraspinatum tendon in rats was changed at different time points after T2DM induction, and the 103 DEGs identified may be potential molecular markers of diabetic tendinopathy. The cAMP signaling pathway and Calcium signaling pathway may be a potential pathogenic pathway for the occur of diabetic tendinopathy. Our work provides complete and detailed data support for exploring the relationship between diabetes and tendinopathy, which is one of the biggest innovations and contributions of this study. Our dataset provides a wealth of differentially expressed genes, each of which provides a basis for future research. The data could be useful to future researchers, giving researchers access to a huge subset of genes. Finally, these data provide raw data for identifying potential biological targets for future targeted therapies.

However, our study still has certain limitations. First of all, these 103 DEGs may play different roles in different stages of the pathogenesis of diabetic tendinopathy, so it is time-sensitive to some extent. However, our study did not carry out more in-depth verification and biological mechanism discussion based on these target genes. Second, due to the inevitable heterogeneity of experimental animals and the limited size of the sample, future studies need to replicate the experiment in parallel controls with larger sample cohorts. Finally, false positive results of some gene expression changes cannot be ruled out. After all, the transcriptomic sequencing method has certain limitations, and more accurate methods are needed to verify the results. At the same time, combining various platforms and multi-omics in further studies is crucial.

## Conclusion

In this study, we established for the first time a biomolecule list of dynamic changes in rat supraspinatus tendon with the progression of diabetes using transcriptomic techniques. The 103 DEGs identified in this study may provide potential molecular markers for exploring the pathogenesis of diabetic tendinopathy, and the 17 major pathways enriched in KEGG may provide new ideas for exploring the pathogenesis of diabetic tendinopathy.

## Data Availability

The datasets generated and/or analyzed during the current study are not publicly available due the confidentiality of the participants’data, but are available from the corresponding author on reasonable requests.

## Abbreviations

T2DM: Type 2 diabetes mellitus
NG: Normal group
DEGs: Differentially expressed genes
GO: gene ontology
KEGG: Kyoto encyclopedia of genes and gnomes
STZ: Streptozotocinn
PBS: Phosphate-buffered saline
